# A Circular Box-Based Deep Learning Model for the Identification of Signet Ring Cells from Histopathological Images

**DOI:** 10.3390/bioengineering10101147

**Published:** 2023-09-29

**Authors:** Saleh Albahli, Tahira Nazir

**Affiliations:** 1 Department of Information Technology, College of Computer, Qassim University, Buraydah 51452, Saudi Arabia; salbahli@qu.edu.sa; 2 Faculty of Computing, Riphah International University, Islamabad 44600, Pakistan

**Keywords:** medical imaging, histopathological images, signet ring cell, CircleNet, DenseNet

## Abstract

Signet ring cell (SRC) carcinoma is a particularly serious type of cancer that is a leading cause of death all over the world. SRC carcinoma has a more deceptive onset than other carcinomas and is mostly encountered in its later stages. Thus, the recognition of SRCs at their initial stages is a challenge because of different variants and sizes and illumination changes. The recognition process of SRCs at their early stages is costly because of the requirement for medical experts. A timely diagnosis is important because the level of the disease determines the severity, cure, and survival rate of victims. To tackle the current challenges, a deep learning (DL)-based methodology is proposed in this paper, i.e., custom CircleNet with ResNet-34 for SRC recognition and classification. We chose this method because of the circular shapes of SRCs and achieved better performance due to the CircleNet method. We utilized a challenging dataset for experimentation and performed augmentation to increase the dataset samples. The experiments were conducted using 35,000 images and attained 96.40% accuracy. We performed a comparative analysis and confirmed that our method outperforms the other methods.

## 1. Introduction

As per a report by the World Health Organization, SRC carcinoma is an inadequately cohesive carcinoma, that is, a combination of large tumor cells with permanent cytoplasmic mucin and a semi-circular-shaped nucleus that is unconventionally located. A total of 90% of SRCs exist in the stomach but are present in smaller numbers in other organs, i.e., the pancreas, colon, and gallbladder [1].

**Figure 1:**
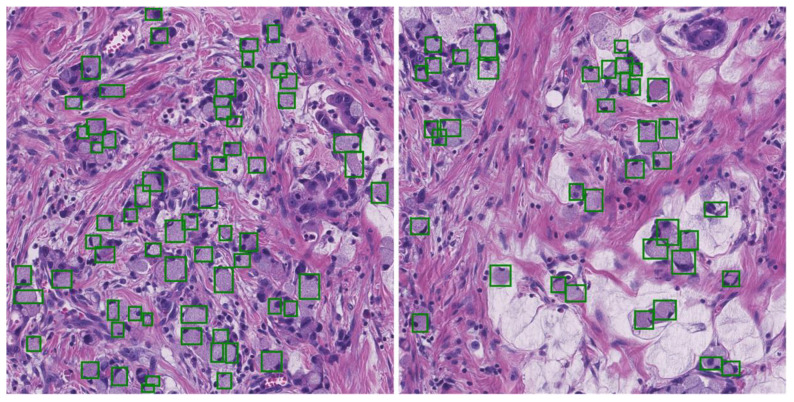
Sample images; the green boxes show signet ring cells in the images.

Ring cell carcinoma can form on its own or in conjunction with any sort of malignant tumor in an organ [2]. The nuclei surrounding ring cells typically have a crushed appearance, and they usually have mucinous cytoplasm. Ring cells are uncommon compared with other kinds of gastric cancer and are easy to miss during microscopic examinations [3]. Atrophic gastritis is difficult to identify from background mucosa, and because it resembles gastritis, untrained endoscopists may fail to make an early diagnosis [4]. Since the stage of the disease impacts the intensity and course of treatment, patient survival rates vary according to cancer stage. Although patients with advanced stomach cancer have a terrible prognosis, patients with an early diagnosis have a five-year survival rate of more than 90% [5]. Since the t-stage is typically used to determine the grounds for endoscopic resection and minimally invasive surgery, the extent of tumor invasion is crucial to determining a patient’s therapy [6]. Therefore, the early detection of SRCs will improve patients’ chances of receiving the right care. Early diagnosis also presents a chance to treat patients with organ-preserving endoscopic procedures like endoscopic mucosal resection [7]. A sample image of SRCs affected by carcinoma is shown in Figure 1.

Medical imaging is essential and frequently used for early cancer detection, monitoring, and post-treatment follow-up [8]. However, manual interpretations of numerous medical images take a lot of time and are prone to errors. As a result, beginning in the early 1980s, computer-aided diagnosis systems were developed to improve productivity and aid physicians in interpreting medical pictures [9].

DL is currently being used in computer-aided diagnosis systems, which outperforms traditional computer vision techniques like machine learning (ML). DL-based artificial intelligence has recently advanced in numerous medical sectors and has proven to be the most successful artificial-intelligence-based analysis in computer-aided imaging [10]. Many different fields have used artificial intelligence for image classification and image detection, including the diagnosis of gastrointestinal neoplasms [11], the comparison of drugs [12], the diagnosis of retinal disease [13,14], and the detection of metaphase [15].

Even though researchers have begun to suggest procedures for the identification of SRCs, it is still difficult to accurately identify these cells, which are associated with hazardous diseases. Moreover, the early recognition of SRCs is a complex task due to their diverse characteristics, such as their shape, size, and color. Machine learning (ML) techniques outperform human brain intelligence in their capacity to handle difficult real-world issues. However, the main problems with ML-based approaches are their low effectiveness and prolonged processing times due to the fact that these frameworks generate long, complex codes that raise computational complexity. Regarding the cost of increased code complexity, DL-based approaches have been developed to address the problem of long codes. Additionally, it is difficult to apply established methodologies to real-world settings.

Due to the intricate appearance of SRCs, the detection of these cancer cells is a difficult task. In the proposed approach, to address the shortcomings of the previous techniques, we introduce a framework using CircleNet with ResNet-34. For the extraction of keypoints, we used the ResNet-34 method, in which the CircleNet framework is used to localize and classify the SRCs. ResNet is a good feature extractor because it has fewer parameters and is also more lightweight than the other models. In addition, we used a challenging dataset with challenging SRC sizes, colors, and forms containing several image artifacts such as noise, blurring, and intensity changes. The primary contributions of our research are listed below:

- We present the CircleNet model, which performs well in a variety of settings including noise, blurred light fluctuations in size, etc.
- Our approach is capable of identifying SRCs and has no trouble identifying photos of a healthy patient.
- The ResNet-34 framework’s accurate feature computation power makes the suggested technique resistant to SRC recognition.
- Because the CircleNet architecture can handle overfitted training data, the provided system is resilient to image post-processing attacks.
- To avoid overfitting, we performed data augmentation, which also increases the model accuracy.

The next part of this study is set up as follows: in Section 2, we present previously published studies. In Section 3, we present investigations on data preparation, data augmentation, and the suggested method. Section 4 includes a discussion of the results, analyzed together with the experimental findings. The conclusion and future work are included in Section 5.

## 2. Related Work

Here, an in-depth analysis of the work already performed for SRC identification and recognition is reviewed. The rapid increase in the evolution of numerous cancers has urged the research community to introduce computer-aided diagnostic systems due to the slow detection ability of manual procedures. Moreover, the existing systems are vastly reliant on the accessibility of domain experts. The extensive progression in the area of AI and ML has introduced powerful approaches that are able to detect medical diseases due to a high recall rate [16,17,18,19,20,21].

The early identification of SRCs is a time-consuming and difficult task because of the complex properties of the cancer cells, which alter their structure from other types of body cells [22]. Several techniques accompanying different ML approaches, including supervised or semi-supervised methods, have been investigated by researchers. However, such methods were found to be not very proficient for the accurate detection of SRCs from medical samples. Li et al. in [23] introduced a methodology to identify and classify SRCs from healthy cells of human bodies using a semi-supervised learning approach. The work presented in [23] shows improved SRC recognition results by merging the techniques that promote the efficient use of both labeled and unlabeled samples. However, this approach is economically inefficient. Similarly, a technique was proposed in [24] where a DL framework was used to locate SRCs in suspected samples by reducing the occurrence of false positives using the partially annotated samples. Specifically, an object detection model named RetinaNet was used to correctly detect and classify the samples either as healthy or SRC-affected. To improve the recognition ability of the introduced approach, the model used a self-learning technique using non-maximum suppression to enhance the detection power of the RetinaNet model. This work showed better SRC detection and classification results; however, the model was unable to perform well under the occurrence of intense brightness changes.

Wang et al. [25] also presented a framework to locate the occurrence of SRCs in the human body by proposing a model, namely, the classification reinforcement detection network (CRDet), based on cascaded RCNN. The major goal of the CRDet approach was to improve the SRC detection performance of the model by selecting a reliable set of keypoints that could assist in locating the affected areas of small size. The approach used in [25] performed well in locating SRCs of small sizes; however, there was an over-fitting issue. Another DL approach was used in [26] to identify SRCs from medical images. The technique described in [26] used the region proposal approach and the embedding model layers, which permitted resemblance learning for model training. The approach was efficient in locating and classifying SRC-affected areas; however, the classification accuracy needs enhancements. Lin et al. [22] used decoupled gradient harmonizing along with classification loss. The approach was proficient in identifying diseased areas; however, performance decreased in the case of noisy samples. Saleem et al. [27] introduced a DL framework to accomplish the recognition of SRCs from medical images. Specifically, the authors used an object detection approach known as Mask-RCNN. Initially, the suspected samples were passed as input to the Resnet-101 module to extract the keypoints, which were later delivered to RPN to produce region RoI proposals along with the use of the RoiAlign unit. Then, the RoIAlign module of the Mask-RCNN model merged the keypoints maps along with the RoI proposals to create segmentation masks. Finally, the fully connected (FC) layer of the model executed the segmentation task by drawing the bonding region over the affected areas and outputting the classification score. The work showed better SRC detection and segmentation results; however, it suffered from the high computational cost.

Another approach was presented in [1] for the automated detection of SRCs. The preprocessed images were used to train several DL approaches like VGG16, VGG19, and InceptionV3. The method attained the best results for the VGG16 model; however, the framework requires extensive training data. Moreover, in [23], a framework using both conventional ML and DL approaches was proposed to facilitate the early diagnosis of SRCs from input images. The method was robust to SRC detection; however, the recognition performance needs further improvements. Zhang et al. [28] proposed an approach for SRC detection and categorization from noisy samples. The authors proposed a DL framework, namely, RetinaNet, combining an unfolding super-resolution network (REUR) to locate the occurrence of SRCs in low-quality samples. In the first step, the model used the super-resolution (SR) unit, which is capable of differentiating high and low-quality samples from the training samples. Then, the approach used the label correction unit to enhance the ground-truth labels from noisy samples, which were later passed as input to the gradient harmonizing mechanism for training loss computation. Finally, the affected region was located with the help of the binding box. The method proposed in [28] was proficient in detecting the SRCs from distorted images; however, it suffered from increased computational cost.

Numerous approaches have been discussed by researchers for the reliable and timely detection of SRCs; however, there is a need for a more efficient approach. Table 1 lists a summary of the existing works. In this study, we tried to fill this gap by proposing a more accurate and effective model for SRC recognition.

## 3. Proposed Method

**Figure 2:**
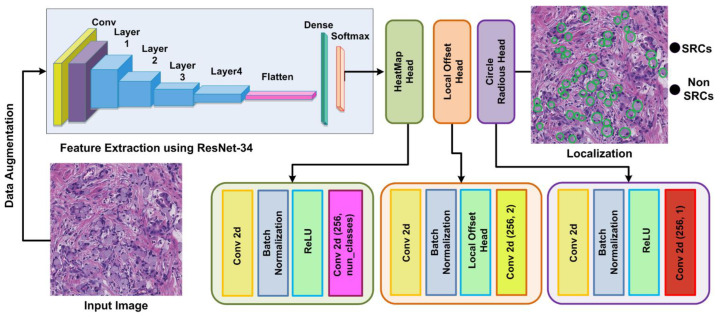
Architectural figure showing the proposed method.

We proposed an improved CircleNet for signet ring cell detection from histological images. The method has the following modules: firstly, annotations are performed and then a localization step is performed to recognize the SRC with the respective class. The overall structure of our methodology is described in Figure 2. According to our proposed method, we generated annotations with the help of experts and ground truths, which are essential for model training. Afterward, in the test module, the input images along with the bounding circle are fed into the model for feature extraction. The Resnet-34 network is used for deep feature estimation from input images. In the final module, the proposed CircleNet is utilized for the localization and classification of SRC. So, the performance of our framework is evaluated using computation metrics that are standard in computer vision. The steps are given in Algorithm 1.

### 3.1. Data Preparation

In this step, we performed data augmentation and also generated annotations of the data. DL-based methods require a lot of data for training, so data augmentation is an essential process to increase the data. Data augmentation can be helpful in attaining a higher accuracy of the model and reducing the overfitting problem. Data augmentation is utilized for the creation of data, which is a useful technique for increasing the quantity and variety of data. In this paper, we applied geometric augmentation approaches, i.e., blur, cropping, flip, and rotation, on the given data to increase its size.

In the next step, we generated annotations with the help of experts and ground truths. Annotations are necessary to recognize the SRC area in the image; for this purpose, we utilized the VGG annotator [29]. The annotated bounding circle values and their respective class details are kept in the file, which is later used for model training. The final annotated file is used as input along with images in the proposed CircleNet framework.
**Algorithm 1:** Steps for the Presented Approach**INPUT:** TS, annotations
**OUTPUT:** Localized RoI, C_Net, Classified lesion   **TS: training samples**   **Annotation (position): bounding box coordinates of lesions in sample**   **Localized RoI**: lesion position   **C_Net-: ResNet34-based CenterNet****Image-Size ← [p q]** **// Bounding box approximation** ***Ӓ* ← AnchorsEstimation (TS, annotation)**
// **C_Net framework**
**C_Net ←** ResNet34-based-CenterNet **(*Image-Size***, *Ӓ***)**
**[T*r*, T*s*] ←** partitioning of the database into ***train* and *test set***
// **Lesion Identification Training Unit**
**For** each sample ***I*** in → ***Tr***
   **Compute** ResNet34 keypoints → n***m*****End**
Training **C_Net** over nm, **and measure training time *t_res***
*η_**res*** ← PreLesionLoc**(**n***m*)**
***Ap_res*** ← **Evaluate_AP*****(ResNet34,** η**_res)***
**For** each sample ***I*** in → ***Ts***
    (**a**) **Compute keypoints using** trained model **€→β*I***;    (**b**) **[*Bounding_box, objectness_ score, class*] ←**Predict **(β*I*)**;    (**c**) **Show sample along with *bounding_box, class***;    (**d**) *η* ← **[***η*
***bounding_box*].** **End For**
**A*p*_€ ← Evaluate** model **€** using *η*
**END**


### 3.2. CircleNet

The efficient and accurate recognition of SRC is challenging and can be completed effectively if the framework computes the deep features of images. However, the computation of deep features is a complex task because of various factors, for example, the model would miss some essential features if it utilized a small set of features. Another issue arises when the model uses a large set of features, which increases the computational time.

Texture-based feature detection techniques are not robust to SRC recognition because of different variations present in color, size, and positions. Moreover, an accurate SRC detection model demands the proposal of a system that is competent and adequate to instinctively acquire image features without the requirement of using hand-crafted keypoints. To address the above issues, we presented a CircleNet model that automatically understands the varying nature of SRC regions from histological images. The CircleNet layers deeply learn the features from test images by exploring their structural details.

The evolution of several object recognition approaches has compelled researchers to use them in medical image analysis. These methods are classified either as two-stage, like the RCNN [30], Fast-RCNN [31], Faster-RCNN [32], or one-stage, like YOLO, SSD, RetinaNet, CornerNet, and CenterNet. The problem with two-stage models is that they offer improved classification solutions at the overhead of an increased processing cost. Two-stage techniques follow two phases to discover and classify suspected regions from the images, which makes them ineffectual for real-world examples. One-stage models offer a low-cost result to SRC recognition but also have low performance gain. Therefore, accurate SRC identification is a complicated task because of some factors including: (i) extensive variations in color, size, and shape and (ii) the existence of intensity fluctuations and the occurrence of multiple SRCs in an image. Furthermore, to handle the above-stated challenges and to address the tradeoff between both SRC identification performance and time, we chose to use the CircleNet model due to its ability to learn important image features while preserving the time complexity as well. Moreover, the cbbox generation ability of CircleNet makes it suitable for SRC identification while maintaining its contours as well.

### 3.3. ResNet-34-Based Features Extractor

All object detection models usually use a CNN framework that is focused on computing the keypoints of input images to extract meaningful information from samples and show it in a viable manner. A computed feature vector is utilized to identify regions of interest (RoI) and accomplish a classification task in numerous object recognition approaches. Descriptively, the more reliable a base model in computing a nominative set of sample features, the more chances there are to enhance the object identification and classification results [33]. For this reason, we selected ResNet-34 [34] together with the convolutional block attention module (CBAM) [35] as the backbone of the improved CircleNet mode, which eventually enhanced SRC recognition performance. ResNet is a well-known DL model that exploits identity shortcut links and residual mapping between the framework layers to attain efficient accuracy. The complex DL models forwarded the results of each earlier layer to the next layer, which calculated a dense keypoints vector. However, for such techniques, the extensive increase in the depth may affect the model recognition performance due to the occurrence of vanishing gradient issues in the model training phase. To address the issues of existing approaches, the ResNet technique proposed the concept of residual blocks (RB) that use skip links in deep models to bypass several layers.

**Figure 3:**
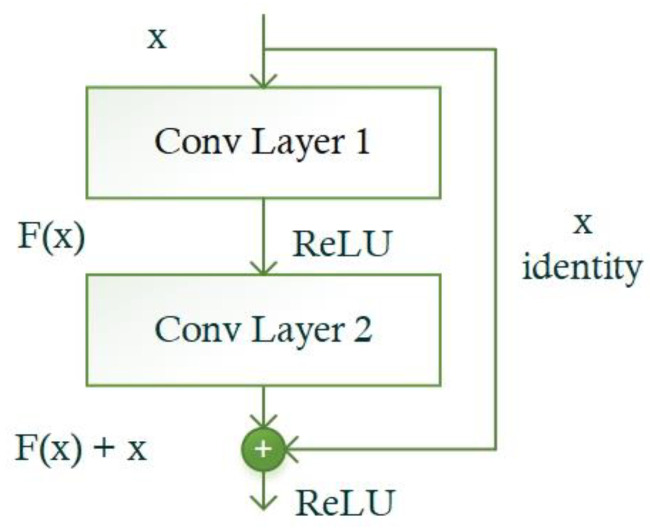
A structural depiction showing RB in the ResNet model.

This structure passes the utilization of computed keypoint maps from the prior layers, which delivers enhanced performance and easier training. A visual depiction showing the RB is given in Figure 3. An RB includes numerous Con layers and uses ReLU as the activation function. Moreover, it encompasses a batch normalization layer together with shortcut connections. Within RBs, the stacked layers are focused on performing the residual mapping by creating shortcut links that accomplish locating mapping (*y*). The outputs are joined with the stacked layers’ output residual function F(y), which is elaborated in Equation (1).

(1) $$Z=M\left(y\right)+y$$

where *y* represents the input, *M* represets the residual method, and *Z* represents the output of the residual method.

We improved the network structure of ResNet-34 by introducing a CBAM-based attention block (AB) [35] at the start of the framework. The basic reason to propose the CBAM block is that it advances the illustration of keypoints using an attention mechanism. The introduced ABs support the model to emphasize the SRC-affected areas while overwhelming unrelated sample data and refining classification results by altering complex circumstances, like chrominance, brightness, and lighting conditions. The CBAM unit upgrades the CNN-computed keypoints by incorporating pixel- and channel-wise attention and consequently boosts DNN results. The CBAM block is trained with the base CNN along with a small added overhead because of its shallow structure. The architectural description of the custom base network is listed in Table 2. Additionally, the starting 7 × 7 Con along with the max-pooling layer are switched with three stacked 3 × 3 Con layers to evade down-sampling phases in the initial Con layer. Moreover, to minimize computational burden, 64 channels are used for newly introduced Con layers.

### 3.4. Heatmap HEAD

After the keypoints are calculated with the help of the modified ResNet34 model, the down-sampling step is used to minimize the feature space. Then, the heatmap head unit takes the extracted feature vector as input to perform a feature approximation. The basic purpose of this module is to locate the SRS-affected areas from the input images. The keypoint approximation results in the computation of the cbbox center to trace the RoI, i.e., SRC regions, which is computed using Equation (2).

(2) $${\hat{o}}_{u,v,s}=\exp(-\frac{{\left(u-\hat{d_{i}}\right)}^{2}+{\left(v-\hat{d_{j}}\right)}^{2}}{2\sigma_{d}^{2}})$$

where *u* and *v* denote the location of the real ground-truth keypoints and $\hat{d_{i}}$ and $\hat{d_{j}}$ denote the location of the computed down-sampled keypoints. Additionally, $\sigma_{d}^{2}$ shows the kernel variance and *s* denotes the total categories, which was two in this study. The ${\hat{o}}_{u,v,s}$ indicates the computed center value over the designated keypoints set having a value up to 1, while the rest are considered as background.

### 3.5. Offset Head

The offset head unit is designated to minimize the discretization error, which usually occurs due to performing the down-sampling phase. After the computation of center points from the heatmaps, they are again shifted to the original sizes.

### 3.6. Dimension Head

The dimension head unit is focused on calculating the coordinates of the cbbox. Once the heatmap peaks are calculated, the next phase is to nominate *H* uppermost peaks having values equivalent to or greater than the eight connected neighbors. A cluster of nominated H central points is calculated using Equation (3).

(3) $$\hat{P}=\left\{\left({\hat{u}}_{k},\;{\hat{v}}_{k}\right)\right\}\;\;\;for\;k=1\;to\;H$$

Lastly, the cbbox is calculated by performing the dot product among the center point $\hat{p}\in \;\hat{P}$ and radius $\hat{r}$. A detailed description of the CircleNet is elaborated in [36].

### 3.7. Multi-Loss Function

To enhance the performance of the proposed CircleNet, we used numerous multi-loss tasks that can successfully identify SRCs having different sizes, colors, and shapes. So, it is important to establish such a model that utilizes efficient loss techniques for accurate SRC detection with classification. The proposed CircleNet used different loss operations at each stage. The usage of numerous losses at each stage of the network allows it to precisely differentiate between the normal and affected regions. The multi-loss L function over each input sample head is given by Equation (4).

(4) $$L_{CN}=L_{h}+\lambda_{r}L_{r}+\lambda_{o}L_{o}$$

where $L_{CN}$ represents the entire loss determined using the proposed CircleNet and $L_{h}$, $L_{r}$, and $L_{o}$ represent the losses estimated across the heatmap, radius, and offset heads, respectively. Moreover, $\lambda_{r}$ and $\lambda_{o}$ are constants having values of 0.1 and 1, respectively. The heatmap loss $L_{h}$ is calculated using Equation (5).

(5) $$L_{h}=\frac{-1}{K}\sum_{u,v,s}\left\{\begin{array}{l} {\left(1-{\hat{o}}_{u,v,s}\right)}^{\alpha }\log({\hat{o}}_{u,v,s}).\begin{array}{c} \;\;\;\;\;\;\;\;\;\;\;\;\;if\;{\hat{o}}_{u,v,s}=1\\ \;\;\;\;\;\;\;\;\;\;\;\;otherwise\; \end{array}\\ {\left(1-O_{u,v,s}\right)}^{\beta }{\left({\hat{o}}_{u,v,s}\right)}^{\alpha }\;\;\;\;\;\;\;\;\;\;\;\;\;\;\;\;\;\;\;\;\;\;\;\;\;\;\;\;\;\;\;\;\;\;\;\;\;\;\;\;\;\;\;\;\;\;\;\;\;\;\;\;\;\;\;\;\;\;\;\;\;\\ \log(1-{\hat{o}}_{u,v,s})\;\;\;\;\;\;\;\;\;\;\;\;\;\;\;\;\;\;\;\;\;\;\;\;\;\;\;\;\;\;\;\;\;\;\;\;\;\;\;\;\;\;\;\;\;\;\;\;\;\;\;\;\;\;\;\;\;\;\;\;\;\;\;\;\;\;\;\;\;\;\; \end{array}\right.$$

where *K* is the total features calculated from the input sample, $O_{u,v,s}$ is the original center of the main feature, and ${\hat{o}}_{u,v,s}$ describes the calculated center of the same main feature. Therefore, *α* and *β* are the hyper-parameters of $L_{h}$ having values of 2 and 4 for all performed investigations, respectively.

Then, the $L_{r}$ is calculated using Equation (6):

(6) $$L_{r}=\frac{1}{K}\sum_{k=1}^{K}\left|{\hat{c}}_{k}-c_{k}\right|$$

where ${\hat{c}}_{k}$ indicates the expected dimensions of the cbbox and $c_{k}$ is the original coordinates of the cbbox taken from the ground truths. Next, $L_{o}$ is calculated using Equation (7).

(7) $$L_{o}=\frac{1}{K}\sum_{d}\left|{\hat{f}}_{\hat{d}\;}-\left(\frac{d}{R}-\hat{d}\right)\right|$$

where ${\hat{f}}_{\;}$ indicates the values of computed offset and d and $\hat{d}$ are the original and down-sampled features.

## 4. Results

This section describes the experimental details including the dataset, evaluation details, results, and comparative analysis of our proposed study. We split our dataset into training and test sets with a ratio of 70:30. The experiments are conducted using a GPU-based machine Intel i7 16 GB ram. To train the proposed model, we choose a learning rate of 0.001, a batch size of 32, and an epoch size of 25. We utilized the Python library TensorFlow for implementation.

### 4.1. Dataset

The DigestPath2019 Grand Challenge competition provided a real public clinical dataset [37] known as the complete slide images SRC dataset, which is used in this work. The dataset includes 90 patient-related samples: 77 positive and 378 negative samples stained with hematoxylin and eosin at a magnification of 9/40. Each negative image sample has a magnification of 2000 × 2000. The dimensions of each positive image sample are comparable but not fixed. SRCs are absent from the negative samples in the dataset. However, pathologists label the SRCs in positive images using annotation techniques.

**Figure 4:**
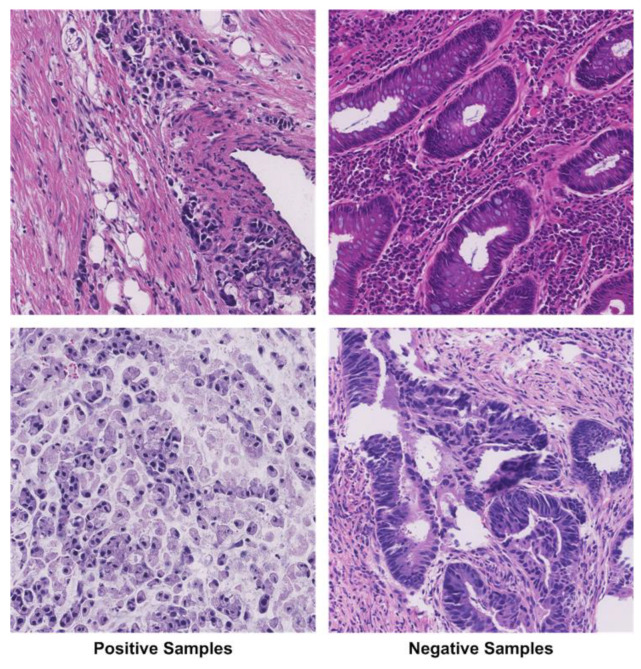
Class samples from the dataset.

The size of the available dataset is 455 images, which is not enough for DL-based model training. So, we applied augmentation to increase the data, and the size of the data after augmentation was 35,000 images. The new data consists of two classes SRC and non-SRC images, as shown in Figure 4: Class samples from the ataset. Additionally, the collection contains some SRCs that pathologists have missed. In the histology sample, it is quite challenging to tell ring cells apart from normal cells.

### 4.2. Evaluation Metrics

For performance measures, we used different parameters, i.e., a confusion matrix (CM), precision, recall, accuracy, F1-score, intersection over union (IOU), and mAP. The CM includes some combinations of predicted and actual values like true positive (TP), true negative (TN), false positive (FP), and false negative (FN) values.

Accuracy is measured by dividing the model’s accurately predicted regions by the total dataset, and the mathematical expression is given in Equation (8).

(8) $$Accuracy=\frac{TP+TN}{TP+FP+TN+FN}$$

Precision is measured by the percentage of positively predicted images that are genuinely positive, as described in Equation (9). It is used to assess classifier accuracy. A low precision rating means that a classifier encounters several FPs. High accuracy values are essential when choosing a model.

(9) $$\mathrm{Precision}=\frac{TP}{TP+FP}$$

Recall displays the proportion of favorable image predictions that occur. A low recall value indicates that the classifier has a high FN rate. For model selection, a high recall value is significant. Equation (10) shows the formula for the recall rate.

(10) $$\mathrm{Recall}=\frac{TP}{TP+FN}$$

The harmonic mean of the precision and recall data is used to obtain the F1 score. The extreme cases should not be disregarded, which is why a harmonic mean should be used instead of a basic one. A model with a precision of “1” and a recall of “0” would have an F1-score value of 0.5, if we assume that the F1-score value is the simple average. This result would be misleading. As a result, the F1-score number includes the harmonic mean calculation. Equation (11) shows the mathematical description of the F1-score.

(11) $$F1-\mathrm{Score}=2\times \frac{\mathrm{Precision}\times \mathrm{Recall}}{\mathrm{Precision}+\mathrm{Recall}}$$

Equation (12) depicts the mathematical formulation of mAP:

(12) $$mAP:=\sum_{i=1}^{T}AP(t_{i})/T$$

where *T* is the number of test images and AP(t_i_) is the average precision of a given test image category. This means that we calculate the AP of each category for a given test image category, *t_i_*, and then the average of each category across all test images. All AP scores would result in a single number, which is mAP, describing how well the trained model is for detection.

IOU is a measure used to indicate how much two boxes overlap. The IOU increases with the size of the overlap region. We calculated IOU using Equation (13).

(13) $$IOU=\frac{\mathrm{Area}\;\mathrm{of}\;\mathrm{Intersection}\;\mathrm{of}\;\mathrm{two}\;\mathrm{boxes}}{\mathrm{Area}\;\mathrm{of}\;\mathrm{Union}\;\mathrm{of}\;\mathrm{two}\;\mathrm{boxes}}$$

### 4.3. Proposed CircleNet Results

In this section, we elaborate on the SRC localization and classification results for the proposed approach using the entire dataset. We performed two types of experiments to show the robustness of our approach. Initially, we performed an analysis to show the SRC recognition power of our model. Then, we discussed the categorization results of our model in classifying the samples either as healthy or SRC-affected with the help of several numerical measures.

### 4.4. Localization Results

**Figure 5:**
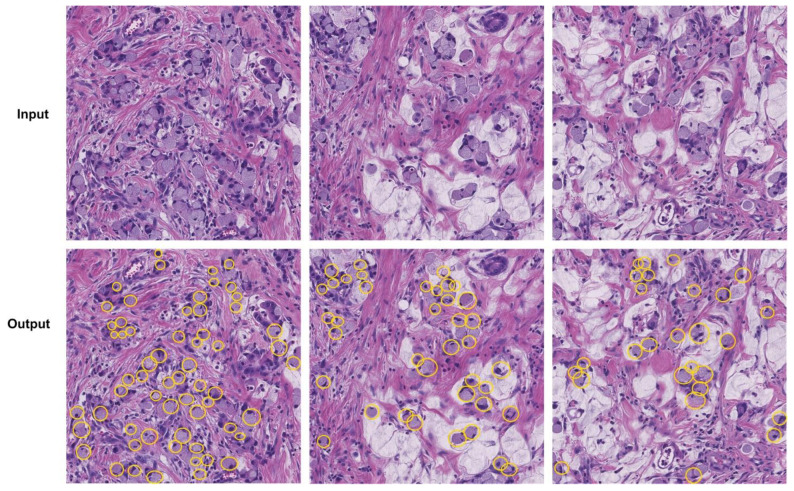
Localization results of the proposed method, where yellow circles show signet rings.

The main characteristic of an SRC recognition model is measured using its ability to correctly locate a diseased region from the input samples. For this reason, we performed an experiment to measure the localization power of the custom CircleNet. The obtained localized results are shown in Figure 5, which indicates that our approach is capable of identifying affected regions of varying sizes, colors, and orientations. Moreover, the proposed approach is capable of preserving the shape of ring cells due to its round binding box generation capability. To numerically assess the power of our model, we used standard measures, namely, mAP and IOU, as these have been heavily explored by scientists using object detection models. The custom CircleNet model attained mAP and IOU scores of 0.959 and 0.961, respectively, which demonstrate the effectiveness of our approach for SRC detection and classification.

### 4.5. Classification Results

In this section, we explain the classification power of our model with the use of numerous standard measures like precision, recall, F1-score, and accuracy.

**Figure 6:**
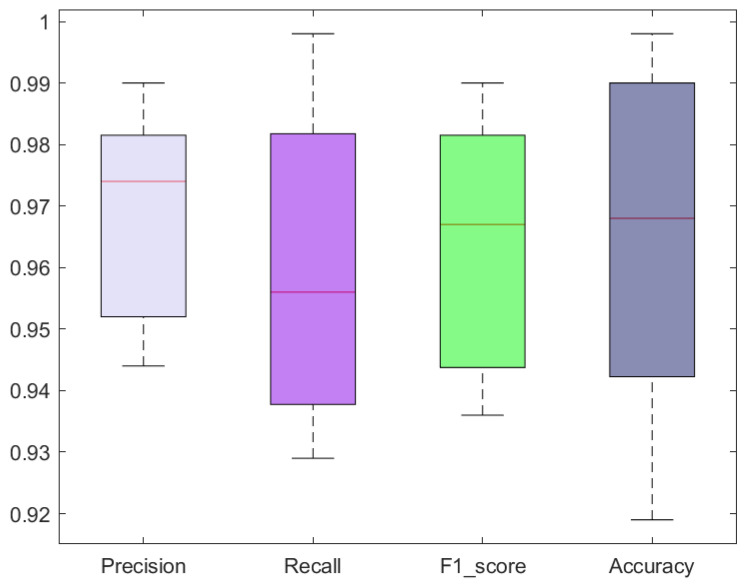
Classification results obtained using the proposed model.

The classification results in terms of precision, recall, F1-score, and accuracy attained with the custom CircleNet for SRC recognition are shown as boxplots (Figure 6), as these provide a better elaboration of results by showing the maximum, minimum, and average obtained values. Specifically, we acquired precision and recall values of 96.80% and 96.10%, respectively. The custom CircleNet approach acquired F1-score and accuracy values of 96.45% and 96.40%, respectively, for SRC recognition, which exhibits the efficacy of the presented model.

**Figure 7:**
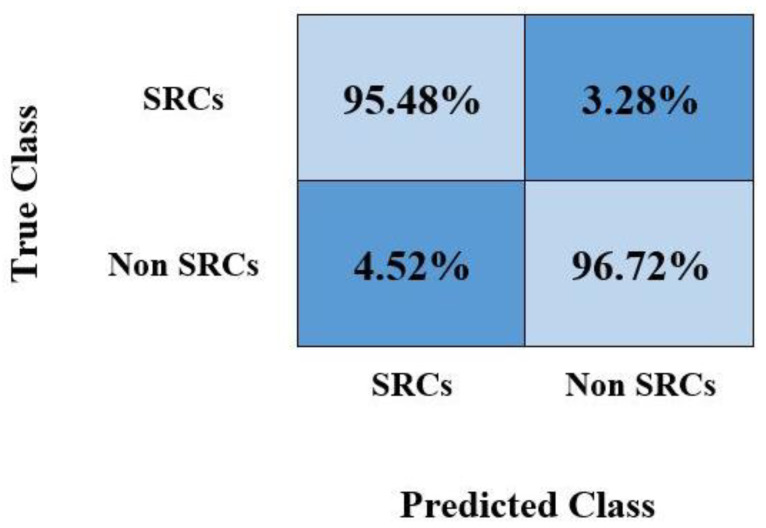
Confusion matrix.

Furthermore, we plotted the confusion matrix as it is the most widely used graph by researchers for discussing classification results obtained by calculating the TPR. The obtained values are shown in Figure 7, which shows that the custom CircleNet model is capable of recognizing healthy and SRC-affected samples accurately.

### 4.6. Proposed System versus Base Models

In this section, we experimentally assessed the SRC classification performance of the custom CircleNet against several base models, namely, Inception-V3, VGG-16, VGG-19, ResNet-101, and GoogleNet. For a fair comparative analysis, we considered the average results obtained using all models over the used dataset and then examined the architectural complexities as well.

Initially, we performed a comparison of our approach with the base models by taking the network structural specifications and discussing the total framework parameters together with their execution time. The results of the analysis are listed in Table 2. The values reported in Table 2 demonstrate that the proposed approach contains a small number of model-trainable parameters and requires less processing time in comparison with the base approaches for SRC detection and classification. Specifically, the VGG19 framework acquires the highest number of model parameters, whereas, in terms of execution time, the GoogleNet model is the most expensive. It is quite evident from the values listed in Table 2 that the proposed custom CircleNet approach outperforms the comparative models in terms of structural complexity as it comprises a small number of parameters and takes minimum time to accomplish the classification task. Based on the values reported in Table 3, it can be said that the introduced model presents a low-cost solution to SRC detection and classification.

**Figure 8:**
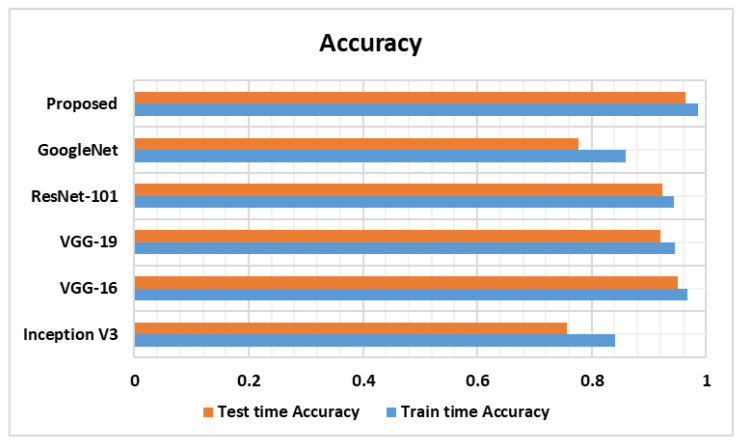
Testing and training accuracy.

Furthermore, we performed a comparative analysis of the proposed approach with the selected models in terms of classification accuracy. The obtained results are depicted in the form of bar graphs (Figure 8). Specifically, InceptionV3, VGG16, VGG19, ResNet-101, and GoogleNet exhibit classification test accuracies of 0.756, 0.951, 0.920, 0.924, and 0.776, respectively. In comparison, the custom CircleNet model attains a classification accuracy value of 0.964, which is higher than all the other approaches. Specifically, the comparative models show an average accuracy value of 86.54%, whereas the average accuracy is 96.40% for our proposed model. Therefore, we achieved a performance gain of 9.86%.

The major cause for the robust SRC detection and classification results of the custom CircleNet model is due to its lightweight structure, which assists in better identifying a more reliable set of feature sets by removing unnecessary data. Such architectural settings of the improved CircleNet model also reduce the total parameters compared with the other DL models, which occupy very deep framework structures and cause the model-tuned issue. Due to this, the comparative approaches are not capable of dealing with several image transformations like the incidence of blurring, noise, and alterations found in the size, chrominance, and brightness of samples. Furthermore, the comparative DL frameworks are not robust enough to tackle unseen cases. The introduced framework has better resolved the limitations of the other approaches by selecting a better set of keypoint vectors that permit the CircleNet model to improve SRC recognition ability. Moreover, the circular bounding box of the CircleNet model allows it to maintain the shape of the detected diseased regions, which further assists in better locating RoIs. Furthermore, the proposed approach minimizes model parameters, which reduces the computational complexity as well. Additionally, the custom CircleNet is capable of generalizing to real-world scenarios due to its better recognition ability. Therefore, we can say that the custom CircleNet approach presents an efficient and robust framework for SRC detection and classification.

### 4.7. Comparison with Object Detection Models

In this section, we performed another experiment to evaluate the proposed approach against other object detection models. Reliable SRC detection and classification is mandatory as a noisy background can deceive the framework when the RoIs have very small sizes. Moreover, the occurrence of several SRCs over a single sample further increases the complexity of the detection procedure. To check this, we considered other object detection models, namely, SSD, YOLO, Fast-RCNN, Faster-RCNN, and Mask-RCNN, and compared our results with results obtained using these models.

To accomplish this, we reported the mAP score and test time for all the selected models, and the attained comparison is listed in Table 3. We calculated the mAP measure and test duration for all models to determine the computational complexity of the performance analysis. An SRC detection comparison of various object detection methods is listed in Table 4. It is evident from Table 4 that in terms of mAP and test time, our strategy outperforms the other approaches. Regarding the mAP values, the SSD, YOLO, Fast R-CNN, Faster R-CNN, and Mask-RCNN approaches achieve mAP values of 0.813, 0.851, 0.845, 0.870, and 0.902, respectively, while our method achieves a mAP value of 0.959 and provides a performance boost of 0.1022. Furthermore, because it produces region suggestions at random and uses the selective search technique, Fast R-CNN is computationally expensive and requires 0.45 s to complete an imaging test. Faster R-CNN processes the suspected sample in 0.31 s and uses the RPN module to automatically generate region proposals while sharing the convolutional layer with the class and bb network to reduce processing costs. SSD and YOLO struggle with small-size SRCs, which reduce their performance. So, our proposed method can detect and recognize small and large-sized objects accurately.

Our method outperforms other methods by creating a bounding circle as well as completing the task in 0.25 s, which is faster than any other comparable method. Based on the results of this study, it is obvious that the proposed approach is more reliable than previous approaches, both in terms of model evaluation and processing time.

### 4.8. Proposed Method versus State-of-the-Art Techniques

For a performance evaluation, we compared our proposed technique with state-of-the-art models proposed by Budak et al. [1], Ying et al. [24], Sun et al. [26], Wang et al. [25], Zhang et al. [28], and Saleem et al. [27]. The reported results obtained using the comparative methods are listed in Table 5. Our method works better than the alternatives. More specifically, we achieved an average accuracy value of 0.964 compared with the other methods, which achieved an average accuracy of 0.91. Thus, we can say that our approach attained a 0.044 performance gain. In the case of precision, the models by Budak et al., Wang et al., and Saleem et al. achieved values of 0.95, 0.428, and 0.901, respectively. Our method attained a higher precision than all other techniques at a value of 0.968. Similarly, for the recall measure, our method yielded an average value of 0.961, which was higher than the comparative approaches. The average recall of the other methods was 0.813 and the performance gain achieved by our method was 0.148, which shows the effectiveness of our method.

The use of CircleNet is much more effective in our case because of SRC shape and region localization. The use of the ResNet-34 framework, which produces a more robust set of image features and helps in improved cancer cell recognition, is the cause of the approach’s successful performance.

## 5. Conclusions

SRC is a dangerous form of cancer that can cause death at the developed stage. The timely recognition of SRCs can save humans from death and painful treatment procedures. Moreover, the complex shape of SRCs also makes the accurate detection of rings a tedious and complex task. We attempted to address the limitations of existing works by introducing a robust framework, namely, the custom CircleNet. Specifically, ResNet-34 was introduced as the backbone network structure of the CircleNet model. Furthermore, the circular bonding box assisted in maintaining the morphological shape of SRCs. Our methods achieved 96.4% accuracy, 0.968 precision, and 0.961 for recall parameters. Both the visual and quantitative values show that our approach can detect and classify SRC regions accurately under the presence of several image distortions. In the future, we will conduct experiments using other datasets and also improve our system performance.

## Figures and Tables

**Table 1:** Summary of related work.

Reference	Method	Results	Limitation
[1]	VGG 16	Recognition of SRCs	Requires extensive training data
[22]	Decoupled gradient harmonizing	Proficient in identifying diseased areas	Performance decreases in the case of noisy samples
[23]	Semi-supervised learning	Classification of SRCs	Economically inefficient approach
[24]	RetinaNet	Binary classification with good results	Unable to perform well under the occurrence of intense brightness changes
[25]	Classification reinforcement detection network (CRDet)	Locates the occurrence of SRCs	Overfitting issue
[26]	Resemblance learning	Efficient in locating and classifying SRCs	Classification accuracy needs enhancements
[27]	Mask-RCNN	Classification and segmentation of SRCs	Small dataset
[28]	RetinaNet	SRCs detection and categorization from noisy samples	Increased computational cost

**Table 2:** Detailed architecture specification of the conventional and modified ResNet-34 model.

Name	Conventional	Improved
Con1	7 × 7, 64, 3 × 3 max pool	$\left[3\times 3,\;64\right]$ × 3, 7 × 7 Attention
Con2_x	$\left[\begin{array}{c} 3\times 3,\;64\\ 3\times 3,\;64 \end{array}\right]$ × 3	$\left[\begin{array}{c} 3\times 3,\;64\\ 3\times 3,\;64 \end{array}\right]$ × 3
Con3_x	$\left[\begin{array}{c} 3\times 3,\;64\\ 3\times 3,\;64 \end{array}\right]$ × 4	$\left[\begin{array}{c} 3\times 3,\;64\\ 3\times 3,\;64 \end{array}\right]$ × 4
Con4_x	$\left[\begin{array}{c} 3\times 3,\;64\\ 3\times 3,\;64 \end{array}\right]$ × 6	$\left[\begin{array}{c} 3\times 3,\;64\\ 3\times 3,\;64 \end{array}\right]$ × 6
Con5_x	$\left[\begin{array}{c} 3\times 3,\;64\\ 3\times 3,\;64 \end{array}\right]$ × 3	$\left[\begin{array}{c} 3\times 3,\;64\\ 3\times 3,\;64 \end{array}\right]$ × 3

**Table 3:** Comparative analysis with the base method.

Parameters	Inception V3	VGG-16	VGG-19	ResNet-101	GoogleNet	Proposed ResNet-34
Total parameters (million)	41.2	119.6	138.3	42.5	6.5	20.3
Execution time (s)	2042	1851	1983	2976	3366	1049

**Table 4:** Comparison with other object detection models.

Method	mAP	Test Time (s/img)
SSD	0.813	0.40
YOLO	0.851	0.27
Fast R-CNN	0.845	0.45
Faster R-CNN	0.870	0.31
Mask-RCNN	0.902	0.29
Proposed CircleNet	0.959	0.25

**Table 5:** Our method versus state-of-the-art models.

Reference	Accuracy	Precision	Recall
Budak et al. [1]	0.95	0.95	0.950
Ying et al. [24]	-	-	0.877
Sun et al. [26]	-	-	0.735
Wang et al. [28]	-	0.428	0.742
Zhang et al. [25]	0.89	-	0.677
Saleem et al. [27]	0.918	0.901	0.897
Proposed CircleNet	0.964	0.968	0.961

## Data Availability

We used standard data, which are publicly available and can be provided upon reasonable request.
